# Research on the influencing factors of users’ information processing in online health communities based on heuristic-systematic model

**DOI:** 10.3389/fpsyg.2022.966033

**Published:** 2022-10-17

**Authors:** Yunyun Gao, Liyue Gong, Hao Liu, Yi Kong, Xusheng Wu, Yi Guo, DeHua Hu

**Affiliations:** ^1^Department of Biomedical Informatics, School of Life Sciences, Central South University, Changsha, China; ^2^Shenzhen Health Development Research and Data Management Center, Shenzhen, Guangdong, China

**Keywords:** online health community, heuristic-systematic model, information processing, influencing factors, information ecology, self-efficacy, privacy trade-off

## Abstract

With the rapid development of the Internet and the normalization of COVID-19 epidemic prevention and control, Online health communities (OHCs) have gradually become one of the important ways for people to obtain health information, and users have to go through a series of information processing when facing the massive amount of data. Understanding the factors influencing user information processing is necessary to promote users’ health literacy, health knowledge popularization and health behavior shaping. Based on the Heuristic-Systematic Model (HSM), Information Ecology Theory, Privacy Trade-Off and Self-Efficacy Theory, we constructed a model of factors influencing user information processing in online health communities. We found that information quality and emotional support had indirect effects on heuristic and systematic information processing, and these effects were mediated by privacy concerns and self-efficacy. In our research model, systematic information processing was most positively influenced directly by self-efficacy. Privacy concerns had a direct negative correlation with both dual information processing pathways. Therefore, OHCs managers should develop relevant regulations to ensure the information quality in OHCs and improve privacy protection services to promote user information processing by improving users’ self-efficacy and reducing their privacy concerns. Providing a user-friendly and interactive environment for users is also recommended to create more emotional support, thus facilitating more systematic information processing.

## Introduction

The Internet has developed rapidly in recent years, and the China Internet Network Information Center (CNNIC) officially released the 48th Statistical Report on the Development Status of China’s Internet. The report shows that as of June 2021, the size of China’s Internet users reached 1.011 billion, an increase of 21.75 million over December 2020, and the Internet penetration rate reached a high of 71.6% (China Internet Network Information Center [CNNIC], 2021). The integration of medical, travel, financial and other industries with the Internet has deepened. At the same time, as people’s quality of life continues to improve, health is gradually becoming a topic of increasing public concern. In the 19th National Congress report, President Xi Jinping put forward the development strategy of “Healthy China,” putting people’s health at the forefront of development and giving them a strategic position., a number of online health communities on the Internet plus medical health model have emerged and flourished, drawing support from policy and technology. At the same time, with the normalization of the prevention and control of the COVID-19 pandemic, online health communities are becoming an essential way for people to obtain health information (Li et al., 2020).

The Online health community is a kind of virtual network community, which is a communication platform established by using Internet technology. In this study online health community is a kind of online platform that allows people to exchange and discuss medical and health information through online, such as Good Doctor Online, Chunyu Doctor, Keep, and other online health communities widely used in China. Users of online health communities include general patients and professional users such as doctors and nurses. We divided OHCs into two categories, a patient-to-physiciancommunity and a patient-to-patient community, Due to our research into the main nature of several online health communities being used in China, we found that one type of online health community is a doctor patient platform, which serves mainly the medical needs of the users, while the other type is a user generated knowledge platform or peer support group social media platform whose main purpose is to exchange information. Different from traditional offline communities, online health communities have no time and space constraints, provide great convenience for people to access health information. Therefore, they played a huge role in improving users’ health literacy (Zhou and Wang, 2020), popularizing health knowledge (Solberg, 2014), and shaping healthy behaviors (Willis and Royne, 2017).

Some studies have pointed out that information content and characteristics significantly impact the attitude, willingness, and behavior of online health community users (Li Y. et al., 2019; Pluye et al., 2019; Peng et al., 2022). Therefore, scholars have made further efforts to track how users’ health behaviors change after acquiring information from online health communities. A study explored the impact of information quality of online health communities on users’ health decisions (Kitchens et al., 2014). Jung et al. (2016) xplored the effect of the credibility of dietary nutrition information websites on user behavior. Social cognitive psychology suggests that a series of information processing processes are required for information to change our behavior (Huijbregts and de Sonneville, 2011). Thus, the user information processing of OHCs deserves further investigation.

In terms of information processing, Li J. et al. (2019) developed a conceptual model of information flow based on the dual information processing paths to study the risk perceptions and behavioral responses affecting Volkswagen customers. Another study explored how motivation and ability affect people’s attitudes toward online health communities in healthcare professionals based on the HSM model (Yada and Head, 2019). However, these studies did not explore the factors influencing user information processing.

When users browse information in online health communities, they go through the processes of information processing such as receiving, encoding, storing, extracting, and transmitting health information (Newell and Simon, 1972). However, most users of online health communities do not have any medical background, and the purpose of this study is to discuss the factors that influence the information processing of OHC users when they are faced with complex health information.

## Theoretical background

### Information dual processing theory: Heuristic-systematic model

Information dual processing theory is one of the important theories in the field of cognitive psychology and social psychology. It expresses how individuals process information and make decisions. It is argued that people have two different modes of thinking. One is a rule-based, rational, controlled, de-contextualized mindset. The other is based on intuition, association, emotion, and highly contextualized; These two modes together act on human behavioral decisions. And the theory of dual processing of information has been developed for many years, forming a variety of theories and models such as Elaboration Likelihood Model (Petty and Cacioppo, 1984), Heuristic-Analytic Model (Evans, 1984) and Heuristic-Systematic Model (Chaiken, 1980), etc. In this study, based on the Heuristic-Systematic Model, the theoretical model is constructed from two paths.

The main features of heuristic processing are intuition-oriented and fast processing, which hardly occupy individual cognitive resources. In contrast, systematic processing is more rationally driven and slower, occupying more cognitive resources in the decision-making process (Trumbo and McComas, 2003). The central feature distinguishing heuristic from systematic processing is whether it requires the participation of working memory resources or cognitive resources. Based on this model, we explored the influencing factors of the two information processing paths.

### Information ecology theory

Information ecology theory was proposed by Hprton (1978), which introduced the viewpoint and concept of ecology into the scope of informatics and emphasized the harmonious coexistence of information subject, information, environment, technology and other factors in the information ecosystem. Among them, the information subject dominates the information activities in the system; information is the object and does not depend on human will; information technology is the carrier of information dissemination; the information environment is the place where the subject and the object interact; the information behavior generated by the information subject and the relationship between the information subject and other elements are the critical issues of the theory.

#### Privacy trade-off

In social exchange theory, it is believed that consumers tend to make decisions based on a cost-benefit trade-off (Wang et al., 2022). The essence of the trade-off view in the information domain is similar to the social exchange theory, which holds that consumers will choose to disclose their personal information as long as the benefits of the act of disclosure outweigh the potential risks associated with the exposure (Culnan, 1995). This trade-off is called the privacy trade-off theory. In a study, privacy is viewed as a person’s (online healthcare platform users) ability to control their health privacy information (Bélanger and Crossler, 2011), and to avoid the potential loss of privacy risk. Users tend to protect their health privacy. Previous behavioral research suggests that individual behavioral intentions are the result of the interaction of individual and contextual factors (Bansal et al., 2016), and that privacy trade-offs are a prerequisite for personal behavioral intentions, which can be influenced by online community factors and user-related factors (Kim and Kankanhalli, 2009).

#### Self-efficacy theory

Self-efficacy, a concept from the theory of planned behavior, is a key determinant in the formation of behavioral intentions and refers to the ability and confidence to successfully complete a behavior (Bandura, 2004). It has been shown that general self-efficacy plays a central role in the self-regulation of motivation and behavior formation in individuals, influences the shape and strength of health promotion intentions, and is one of the best predictors of health promotion intentions (Wang et al., 2006). Conceptually, the higher the level of self-efficacy in general, the stronger the health promotion intention (Sirois, 2015). It has also been shown that the most important influencing factor of individual health information seeking behavior is self-efficacy, and higher self-efficacy promotes individual health information behavior. (Cui, 2022)

Thus, in this study, we combined the Information Ecology Theory, HSM, Privacy Trade-Off and Self-Efficacy Theory to develop a theoretical model to identify the factors influencing users’ information processing in online health communities.

## Research model and hypotheses

### Five elements of information ecology

According to the Information ecology theory, the harmonious coexistence and mutual influence of information subject, information, environment, and technology factors in the information ecosystem.

This study attributes the quality of health information users perceives in the OHCs to the information element. More and more Internet users use online health communities to obtain health care information to help them achieve the goal of health management (Dart and Cindy, 2010; Elwyn et al., 2013). Information quality is a fundamental factor in the validity assessment of information recipients in a systematic process (Gorla et al., 2010). It includes the quality of information content (completeness, accuracy, clarity, etc.) and the quality of the medium (convenience, timeliness, security, accessibility, etc.) (Eppler, 2006). Several studies have shown that users’ perceived information quality enhances their willingness to share knowledge (Kulkarni et al., 2006; Zheng et al., 2014). And service providers can improve the perceived quality of the information services they provide to offset users’ privacy concerns (Li and Unger, 2012). In addition, studies have shown that there is a correlation between online health information and the self-efficacy of online users (Bass et al., 2006).

Therefore, two hypotheses are formulated as follows:

H1: Users’ perceived information quality negatively influences users’ privacy concerns.

H2: Users’ perceived information quality positively influences users’ self-efficacy.

Users’ emotional support and privacy concerns in the OHCs are attributed to the information environment elements. Emotional support is a positive information atmosphere. A good interaction atmosphere strongly motivates users to engage in continuous information-sharing behavior. Users discuss and communicate on the Internet to obtain specific social support, including emotional, informational, companionship, and materialistic support (Hajli, 2014). As a result of the emotional support they receive in OHCs, users feel loved, cared for, and more willing to participate in the community (Zhou and Wang, 2020). A study found that the users’ perceived social support has a negative effect on the relationship between perceived health information sensitivity and privacy concerns (Dang et al., 2021). In addition, emotional support may positively affect users’ self-efficacy (Chair et al., 2016; Al-Dwaikat et al., 2021).

Thus, the following hypotheses were made:

H3: Users’ perceived emotional support negatively influences users’ privacy concerns.

H4: Users’ perceived emotional support positively influences users’ self-efficacy.

And user-perceived system quality is attributed to information technology elements. A study of persistent use by users of online knowledge communities found that the system quality of knowledge communities significantly influenced users’ intention to persist and users’ consistent use behavior (Zhou, 2018). The platform design of the OHCs brings users a better experience when obtaining information by providing a beautiful and comfortable experience (Young, 2013). The ease of use of a platform positively impacts users’ willingness to use it (Salahshour et al., 2016), and the stability of information systems is essential to web users (Delone and Mclean, 2003). A high-quality system environment facilitates users into the group interaction atmosphere and alleviates user privacy concerns (Zhou, 2016). At the same time, it was found that people’s self-behavior ability results from the interaction of subject (person), behavior, and environment (Bandura, 1977). There is a correlation between systemic environment and self-efficacy.

Thus, we posed the following hypotheses:

H5: Users’ perceived system quality negatively influences users’ privacy concerns.

H6: Users’ perceived system quality positively influences users’ self-efficacy.

A negative information atmosphere is created by users’ privacy concerns. The endless privacy leaks are the consequence of the rapid development of Internet technology. Users of the OHCs who perceive information privacy risks when sharing information and knowledge will be discouraged from interacting and actively hide some personal information (Hajli and Lin, 2016). Privacy concerns influence users’ attitudes toward information disclosure; the more concerned users are about privacy issues, the more negative their willingness to disclose information (Bansal et al., 2010). Therefore, the following hypotheses were proposed.

H7: Users’ privacy concerns negatively influence their heuristic information processing.

H8: Users’ privacy concerns negatively influence their systemic information processing.

Users’ Self-efficacy is attributed to the information subject element of the OHCs. Self-efficacy refers to an individual’s perceptions or beliefs about their ability to behave adaptively in the face of challenges in their environment (Bandura, 1977). In our study, self-efficacy refers to the perceived ability of online health community users to collect, analyze, and use a range of information processed within the OHCs. There is a mutually reinforcing relationship between self-efficacy and the ability to use information, and self-efficacy plays an essential role in the individual’s information processing (Moriyama et al., 2009; Lam et al., 2012). Therefore, we established the following hypotheses:

H9: Users’ Self-efficacy positively influences their heuristic information processing.

H10: Users’ Self-efficacy positively influences their systemic information processing.

**FIGURE 1:**
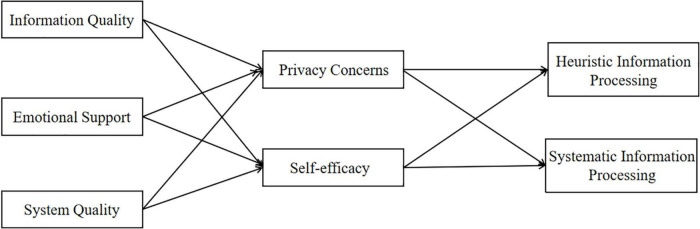
Structural research model.

We summarized our hypotheses in Figure 1.

## Materials and methods

### Development of the study questionnaire

A cross-sectional study was conducted and a questionnaire was developed for the research model to collect data composed of demographic characteristics, information about the user’s use of the OHCs and the measurement items for each latent variable. The questionnaire items (Table 1) were measured with a 5-point Likert scale ranging from “strongly disagree” (1) to “strongly agree” (5).

**TABLE 1:** Measurement items of the constructs.

Constructs	Measurement items	Source
Information quality (IQ)	I think the health information provided by the OHCs is useful.	Wixom and Todd, 2005; Yi et al., 2013
	I think the health information provided by the OHCs is accurate.	
	I think the health information provided by the OHCs is timely.	
Emotional support (ES)	Other users in the OHCs have provided me with encouragement and support.	Oh et al., 2013
	Other users in the OHCs have given me sympathy.	
	Other users in the OHCs make me feel relaxed.	
Privacy concerns (PC)	The OHCs do not adequately protect my privacy.	Zhou, 2018
	Sharing information through online health communities can compromise my privacy.	
	I think a third party (such as a hacker) will steal and reveal my privacy.	
Self-efficacy (SE)	I can get the health knowledge and experience I want from the OHCs.	Kim et al., 2009
	I can better understand and use the health information provided in the OHCs.	
	I can communicate and share health information with other users in the OHCs in a timely and effective manner.	
System quality (SQ)	I think the OHCs are designed to be beautiful and comfortable.	Lin, 2006
	I think the OHCs are convenient and easy to use.	
	I think the OHCs are stable and not easy to crash.	
Heuristic information processing (HIP)	When using the OHCs, I search for health information by feeling.	Smerecnik et al., 2012; Tortosa-Edo et al., 2014; Li J. et al., 2019
	When using the OHCs, I find useful information and then stop searching for other relevant information.	
	When using the OHCs, I won’t spend much time thinking about health information.	
	When using the OHCs, I do not evaluate the quality of health information.	
	When using the OHCs, I don’t think twice about adopting the health information I find.	
Systematic information processing (SIP)	When using the OHCs, I spend a lot of effort searching for relevant health information.	
	When using the OHCs, I find myself making connections between the health information and what I’ve read or heard about elsewhere.	
	When using the OHCs, I think about how the health information relates to other things I know.	
	When using the OHCs, I correlate the information I get with my health status.	
	When using the OHCs, I think about applying the information I get to my health decisions.	

The presurvey sample size was often suggested to be 5–10 times the number of observed variables of the scale, greater than or equal to 100 (Anthoine et al., 2014). Reliability was measured using Cronbach’s alpha. In our presurvey analysis, the questionnaire was sent to 123 users of OHCs for a presurvey in July 2021.

The Cronbach alpha of all constructs should be higher than 0.70 (Martynova et al., 2018). We used exploratory factor analysis with varimax-rotated components to measure the validity of the designed questionnaire, and its cumulative total of variance and factor loadings were used to assess the construct validity. If the cumulative total variance of the principal components selected accounted for more than 70% of the total variance, the composition of the principal components as exogenous constructs was consistent with the constructs of the designed questionnaire, and if each item had a factor loading value of 0.50 or higher on one of the principal components but factor loading values below 0.50 on others, the validity of the designed questionnaire was considered acceptable, and no items were removed.

### Data collection

The formal questionnaire was conducted electronically and distributed to the OHC users through the question and answer section of WeMed and Haodaifu, the health forum bar of Baidu, Zhihu, and the health support groups on QQ and WeChat. The questionnaires were distributed from July 2021 to September 2021, and we received a total of 347 questionnaires. Of these, 20 responses were excluded because they had never used an online health community, contained more than five consecutive questions with the same options, displayed certain logical contradictions, duplicate IP addresses, or because the time taken to answer the questionnaire was less than 60 s. Three hundred twenty-seven valid samples were retained, and the sample validity rate was 94.23%.

### Statistical analysis

We used structural equation modeling (SEM) to verify the research model. Before evaluating the structural model, Cronbach alpha and composite reliability (CR) were used to measure the reliability. All constructs were considered acceptable with a Cronbach alpha value of 0.70 or higher and a CR value of 0.7 or higher (Zhang et al., 2019). The average variance extracted (AVE) and standardized loadings were used to measure convergent validity (Lu et al., 2019). The AVE of each construct and all the standardized loadings should be greater than 0.50 (Dou et al., 2017; Hoque and Sorwar, 2017). The model fit was generally considered acceptable when the indexes met the criteria, including χ^2^/df < 3, root mean square error of approximation <0.08, goodness-of-fit index (GFI) > 0.90, comparative fit index(CFI) > 0.90, Tucker-Lewis index (TLI) > 0.90, and normal fit index (NFI) > 0.90 (Dou et al., 2017). Otherwise, the model needs further revision. Data analysis was performed using SPSS.26 and Amos.24 software.

## Results

### Sample characteristics

The demographic characteristics of the 327 participants are shown in Table 2. Nearly half of them have a bachelor’s degree, with a percentage of 56.3. Female participants (199) were higher than male participants (128). Among them, 208 (63.6%) survey respondents were in good health, and only 8.9% of respondents were in poor health. And 68.5% of respondents have no medical background. The age level of the survey respondents was more evenly distributed between 18 and 55 years old, and the number of 46–55 years old was higher, accounting for 27.5%. In terms of frequency of use, we divided them into daily, frequent and occasional users, with the highest number of occasional users, accounting for 62.4%. We also investigated the topics users are more concerned about when using OHCs. Of the 327 respondents, the number of respondents who were interested in disease prevention topics was 153(46.8%), 211 respondents(64.5%) were interested in disease treatment, and 233 (71.3%) respondents were interested in fitness and weight loss topics. The effects of three variables (gender, medical background and OHCs type usage preferences) on Heuristic Information Processing (HIP) and Systematic Information Processing (SIP) were tested by independent sample *t*-test, and the effects of four variables (age, health condition, frequency of using the OHCs and educational background) on HIP and SIP were tested by T-test or OnewayANOVA.

**TABLE 2:** Demographic characteristics of the participants (*N* = 327).

Measure and category	Value (*N* = 255), *n* (%)	*P-value* (HIP)	*P-value* (SIP)
**Gender**			
°Male	128 (39.1)	0.15	0.970
°Female	199 (60.9)	2	
**Health condition**			
°Poor health condition	29 (8.9)	***	0.003**
°General health condition	90 (27.5)		
°Good health condition	208 (63.6)		
**Age in years**			
°<18	2 (0.6)	***	0.014*
°18–25	72 (22.0)		
°26–35	80 (24.5)		
°36–45	67 (20.5)		
°46–55	90 (27.5)		
°>55	16 (4.9)		
**Educational background**			
°Junior College and below	73 (22.3)	0.022*	0.90
°Undergraduate	184 (56.3)		
°Postgraduate and higher	70 (21.4)		
Frequency of using the OHCs			
°Occasionally	204 (62.4)	0.498	0.054
°Frequently	73 (22.3)		
°Daily	50 (15.3)		
**Medical background**			
°Yes	103 (31.5)	***	0.003**
°No	224 (68.5)		
**OHCs Type Usage Preferences**			
°Patient-to-physician type OHCs	166 (50.8)	***	0.216
°Patient-to-patient type OHCs	161 (49.2)		
**Concerned health knowledge topics**			
°Disease prevention	153 (46.8)	–	–
°Disease treatment	211 (64.5)		
°Fitness and weight loss	233 (71.3)		

HIP, heuristic information processing; SIP, systematic information processing.

*P < 0.05; **P < 0.01; ***P < 0.001.

Analysis of independent sample *t*-test and ANOVA suggested that there are statistically significant differences in HIP between groups with respect to health condition, age, educational background, medical background, and OHCs type usage preferences. As for SIP, there are statistically significant differences between groups concerning health condition, age, and medical background.

### Measurement model testing

Table 3 shows the reliability of the questionnaire validated factor analysis carried out with the help of AMOS24. 0 software. As can be seen from the table, the standardized loadings of all indicators are greater than 0.7, the AVE of each variable is greater than 0.5, and the CRs are greater than 0.7, which means that the scale has good convergent validity.

**TABLE 3:** Statistical results of the research model.

Constructs	items	Standard loadings	Cronbach alpha	AVE	CR
Information Quality (IQ)	IQ1	0.877	0.833	0.782	0.915
	IQ2	0.897			
	IQ3	0.875			
Emotional support (ES)	ES1	0.899	0.821	0.797	0.922
	ES2	0.903			
	ES3	0.875			
Privacy concerns (PC)	PR1	0.920	0.854	0.780	0.912
	PR2	0.911			
	PR3	0.872			
Self-efficacy (SE)	SE1	0.908	0.786	0.758	0.904
	SE2	0.880			
	SE3	0.823			
System quality (SQ)	SQ1	0.891	0.829	0.812	0.928
	SQ2	0.909			
	SQ3	0.855			
Heuristic information processing (HIP)	HIP1	0.819	0.816	0.743	0.935
	HIP2	0.849			
	HIP3	0.917			
	HIP4	0.905			
	HIP5	0.813			
Systematic information processing (SIP)	SIP1	0.817	0.831	0.768	0.943
	SIP2	0.871			
	SIP3	0.902			
	SIP4	0.906			
	SIP5	0.883			

CR, composite reliability; AVE, average variance extracted.

The obtained sample data were tested for discriminant validity to verify the degree of distinction between the latent variables. The values on the diagonal in Table 4 are the square root of AVE corresponding to the seven latent variables. The rest of the values are the degree of correlation among the latent variables. The results show that the correlation coefficients of the seven latent variables are smaller than their square root of AVE, indicating a high discriminant validity among the latent variables in the model. And as shown in Table 5, all fit indexes are in the range of the recommended values, indicating that the research model fits the data collected well.

**TABLE 4:** Correlation matrix (*N* = 327).

	IQ	ES	PC	SE	SQ	HIP	SIP
IQ	0.781						
ES	0.703	0.792					
PC	0.718	0.733	0.819				
SE	0.749	0.730	0.633	0.743			
SQ	0.729	0.721	0.662	0.657	0.771		
HIP	0.503	0.503	0.579	0.544	0.454	0.787	
SIP	0.581	0.575	0.591	0.693	0.518	0.437	0.742

IQ, information quality; ES, emotional support; PC, privacy concerns; SE, self-efficacy; SQ, system quality; HIP, heuristic information processing; SIP, Systematic information processing.

**TABLE 5:** Goodness-of-fit results of the revised research model.

Fit indexes	X^2^/DF	GFI	TLI	CFI	RMSEA
Recommended value	<3	>0.8	>0.9	>0.9	<0.08
Research model	2.241	0.872	0.953	0.959	0.062

GFI, goodness-of-fit index; TLI, Tucker-Lewis index; CFI, comparative fit index; RMSEA, root mean square error of approximation.

### Structural model testing

**FIGURE 2:**
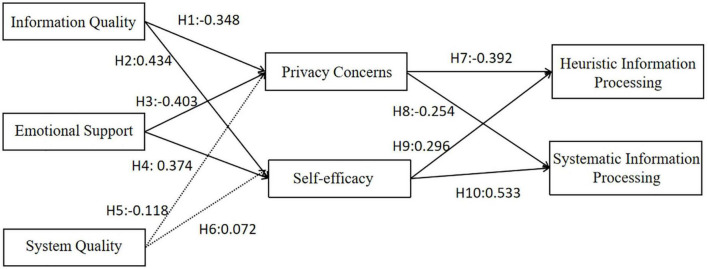
Research model of hypothesis testing.

As shown in Table 6 and Figure 2, two (H5 and H6) of the ten research hypotheses were rejected. Other relationships’ standardized path coefficients were significant at *P* < 0.05. Information quality (IQ) and emotional support (ES) both had a direct negative effect on privacy concerns (PC), and the effect of ES on PC (β = −0.403) is greater than the effect of IQ on PC (β = −0.348). H1 and H2 were supported. Both IQ and ES also had moderate direct positive effects on self-efficacy (SE) (β = 0.434, and β = 0.374, respectively). H3 and H4 were supported. Additionally, PC moderate affects, both HIP and SIP negatively (β = −0.392, and β = −0.254, respectively). H7 and H8 were supported. It is thus evident that HIP is slightly more influenced by PC than SIP. Further, SE had a moderate positive direct on HIP(β = 0.296). And SE had a strong effect on SIP (β = 0.533). The effect of SE on SIP is greater than that of HIP. H9 and H10 were supported. The effects of system quality (SQ) on PC and SE were not significant at a *P*-value 0.05 level. H5 and H6 were not supported.

**TABLE 6:** Hypothesis testing results of the research model.

Hypothesis paths			Standardized path coefficients	*P-value*	Results
IQ	→	PC	–0.348	***	H1supported
IQ	→	SE	0.434	***	H2supported
ES	→	PC	–0.403	***	H3supported
ES	→	SE	0.374	***	H4 supported
SQ	→	PC	–0.118	0.098	H5 not supported
SQ	→	SE	0.072	0.315	H6 not supported
PC	→	HIP	–0.392	***	H7 supported
PC	→	SIP	–0.254	***	H8 supported
SE	→	HIP	0.296	***	H9 supported
SE	→	SIP	0.533	***	H10 supported

IQ, information quality; ES, emotional support; PC, privacy concerns; SE, self-efficacy; SQ, system quality; HIP, heuristic information processing; SIP, systematic information processing.

***P < 0.001.

Further, IQ and ES had indirect effects on both HIP and SIP, and these effects were mediated by PC and SE. The indirect effects of IQ and ES on SIP (β = 0.319, β = 0.301) is slightly higher than the effects on HIP (β = 0.265, β = 0.269). Table 7 shows the total, direct, and indirect effects (standardized path coefficients) of the model variables (constructs) on heuristic information processing and systematic information processing, respectively.

**TABLE 7:** Total, direct, and indirect effects of the model variables.

Variables	HIP(ß)	SIP(ß)
**IQ**		
**Direct**		
Indirect	0.265	0.319
Total	0.265	0.319
**ES**		
Direct		
Indirect	0.269	0.301
Total	0.269	0.301
**SQ**		
Direct	−	–
Indirect	−	–
Total	−	–
**PC**		
Direct	–0.392	-0.254
Indirect		
Total	–0.392	-0.254
**SE**		
Direct	0.296	0.533
Indirect		
Total	0.296	0.533

## Discussion

Our research found that there is a certain correlation among the five information elements under information ecology, and information subject and unfavorable information environment have a direct effect on the OHC users’ information processing. Further, IQ and ES had indirect effects on HIP and SIP, which were mediated by PC and SE.

Privacy concerns had a direct negative effect on information processing (both HIP and SIP). With the rapid development of health information technology and the popularity of online health communities (Deng et al., 2018), patients are increasingly concerned about protecting their personal information, such as basic user registration information and their medical information. Some studies have also shown that users’ privacy concerns negatively influence their information behavior (Sun et al., 2019; Yu et al., 2020). When users have privacy leakage concerns about the OHCs, they may prefer offline access to medical care instead of choosing the OHCs to obtain information, thereby refusing to process information online. Furthermore, compared to SIP, PC has a higher degree of negative effect on HIP. This may own to the reason that privacy concerns can cause negative emotions in users, and HIP is more susceptible to its own emotions (Trumbo and McComas, 2003). Therefore, it is necessary to develop further and improve privacy protection standards and regulations for OHCs to guarantee the privacy and security of OHC users and foster a good information processing environment for users by facilitating the information processing behavior of OHC users.

Compared to IQ and ES, SE had a deeper positive effect on SIP than HIP. Users’ health knowledge reserves, information-seeking ability and communication ability are closely related to information processing. The stronger their self-efficacy, the higher their ability level (Lucidi et al., 2006), and the more inclined they are to adopt systematic information processing and take up more cognitive resources when processing information, requiring individuals to think deeply. Therefore, while improving information services on the platform, Healthcare organizations may provide multifaceted support to users with a high level of expertise in healthcare so that they can make the most of that expertise and further build high-quality health information resources.

Meanwhile, an effective incentive mechanism is recommended to be set up to encourage users to create and deeply reprocess health care information resources on top of existing health information resources (Zhiyi et al., 2020). For ordinary users, administrators of OHCs are suggested to offer relevant health knowledge lectures further to enhance the platform’s specialized health information exchange, promote the health information literacy of ordinary users, improve their health knowledge reserves, communication skills and information screening ability to promote their deeper understanding and processing of information.

The indirect positive effect of IQ on both SIP and HIP was slight (and this effect was mediated by PC and SE). The core service of the OHCs is to deliver health information to users (Peng et al., 2019). When users perceive that health information is useful, accurate and timely, they understand and evaluate their health situation correctly, relieve their anxiety caused by their health problems and create a sense of dependence and security to online health communities. As a result they are more willing to trust the OHCs to protect the privacy and security of their personal information (Lu et al., 2018), and build a certain amount of self-confidence in themselves (Anderson et al., 2011).

Therefore, OHCs managers need to improve community information quality and establish specific rules for information review (Wang et al., 2021). For patient-to-physician OHCs, it is important to review and screen information from the original source, and verify the identity of professional users registered on the platform (i.e., doctors, nurses, and other professionals) to further improve the professional accuracy of health information. For patient-to-patient OHCs, there are not only professional users sharing health information but also ordinary users sharing their experiences for other users to communicate and analyze. Therefore, OHC managers should ensure that the health information shared by users on the platform is checked and filtered, so as to deal with false or harmful information on time, thereby reducing the risk of information exposure for users.

Additionally, we also found that ES indirectly influenced the OHC users’ information processing through PC and SE. When users feel more emotional support in the OHCs, the community platform will become more and more familiar to them as they become emotionally attached to it. (Zhao et al., 2013), thus weakening privacy concerns about online health communities and creating trust in the community.

In a good information interaction atmosphere, users are more willing to actively and continuously participate in information activities (Song et al., 2018) and enhance their sense of self-efficacy. Therefore, online health communities need a good communication atmosphere to increase the activity of user interaction and enhance user stickiness. Users’ browsing records can be accurately analyzed to push the content they need and are interested in with the intention of promoting the exchange of information. For patient-to-physician OHCs, patients seek help from doctors and hope to get emotional support (Xue and Chang, 2022). The platform should set up a patient evaluation mechanism and provide feedback to doctors through patient comments, which makes doctors’ services more humane and patients can gain more emotional comfort and trust in online health communities. For patient-to-patient OHCs, certain incentives can be taken to guide users to exchange information and enhance their sense of belonging to the community, so that users can develop emotional dependence on the community and truly integrate into it.

For OHCs users focused on getting information support (e.g., disease knowledge, medical advice, health information) (Young, 2013) to manage their health problems, users pay more attention to the content of the information rather than the external quality of the information, thus to some extent weakening the influence of website system quality on information processing behavior.

## Limitations and directions for future research

This study combines the information ecology theory with the heuristic-systematic model of information dual processing, which provides a new perspective for user information processing research. However, our study still has some limitations. Firstly, As a result of the objective and subjective conditions in this study, the selection of influencing factors is not well considered. Secondly, the questionnaire sample source is limited. Finally, the use of questionnaire methods to measure user information processing is too subjective. In the follow-up study, we intend to expand the reference range of influencing factors to improve the scientificity of the selection of influencing factors and ensure their comprehensiveness. In addition, the health status of the respondents may be a confounding factor, which was not investigated in depth in our study. In future studies, the health status of the respondents could be further explored. With the segmentation of user needs, more different types of online health communities have been derived and tend to diversify, but our study only classifies online health communities into two types, and the behavior of information processing can be further explored in future studies for platforms with different characteristics. As for the exploration of user information processing, we can consider using certain instruments, such as eye-tracking instruments, to reflect the degree of user information processing objectively. Furthermore, the sample scope can be expanded to include different populations in future studies.

## Conclusion

In this study, we established a model of factors influencing the information processing of online health community users based on the HSM model and the information ecology theory. Variables of this research model includes information quality, emotional support, system quality, privacy concerns, self-efficacy, heuristic information processing and systemic information processing. We designed and distributed questionnaires to collect data. The results of our study showed that information quality and emotional support cannot be predicted directly on information processing, and they indirectly influenced online health community users’ information processing through privacy concerns and self-efficacy. Both information quality and emotional support had a negative influence on privacy concerns and a positive influence on self-efficacy. Both privacy concerns and self-efficacy have direct effects on information processing of online health community users, and users’ privacy concerns negatively affect their information processing, while users’ self-efficacy positively affects their information processing. System quality also cannot be predicted directly on users’ information processing.

Therefore, the OHCs managers should further optimize the design of online services, ensure a reasonable assembly of highly qualified medical experts, make reasonable coordination between online and offline services, and continue to deepen the quality of personalized information services, which is conducive to enhancing users’ goodwill toward online health communities and promoting further processing of information. In terms of privacy protection for users, it is not only necessary to establish reasonable privacy protection rules, but also to create a good interactive environment for users and enhance their sensory emotions and pleasant experiences. These immersive experiences can reduce users’ privacy concerns in the process of health privacy self-disclosure and promote positive health information contribution behaviors.

## Data availability statement

The original contributions presented in this study are included in the article/supplementary material, further inquiries can be directed to the corresponding authors.

## Ethics statement

The studies involving human participants were reviewed and approved by Institutional Review Board of College of Life Sciences, Central South University (Reference No:2021-1-45). Written informed consent to participate in this study was provided by the participants or their legal guardian/next of kin.

## Author contributions

YYG, YG, and DH designed the study. YYG and DH led the investigation, formal analysis, and wrote the first version of the manuscript. LG and HL reviewed and edited the manuscript. YK and XW organized and helped revise the manuscript. DH and YG were responsible for the administration of the project. All authors have read and agreed to the published version of the manuscript.
